# Parent and Child Predictors of Internalizing and Externalizing Symptomatology during COVID-19

**DOI:** 10.3390/children10101625

**Published:** 2023-09-29

**Authors:** Simona Scaini, Marcella Caputi, Ludovica Giani

**Affiliations:** 1 Department of Psychology, Sigmund Freud University, Ripa di Porta Ticinese 77, 20143 Milan, Italy; s.scaini@milano-sfu.it (S.S.); giani.phd@milano-sfu.it (L.G.); 2 Child and Adolescent Unit, Italian Psychotherapy Clinics, Corso San Gottardo 5, 20136 Milan, Italy; 3 Department of Life Sciences, University of Trieste, Via E. Weiss 21, 34128 Trieste, Italy

**Keywords:** child, COVID-19, parenting, resilience, psychopathology

## Abstract

The COVID-19 emergency has fostered an increasing risk of experiencing distress and negative emotions in parents that turned into heightened stress for children. In this study, we aim to evaluate the effects of parental stress, children’s resilience, and previous adversities on the development of internalizing and externalizing symptoms in children. A series of questionnaires were completed by 158 Italian parents (148 mothers, 10 fathers, mean age = 41 years) concerning them and their school-aged children (N = 158, 76 boys, mean age = 7.4 years) at two critical time points (June 2020 and December 2020). Regression analyses showed that internalizing problems were predicted only by concurrent children’s resilience, whereas externalizing problems were predicted by concurrent parental flooding, children’s resilience, and early parental satisfaction. Therefore, internalizing and externalizing symptoms trajectories follow different routes and are predicted by both common and distinct factors. Supporting positive parenting attitudes and behavior should be recommended to prevent the worsening of children’s externalizing behaviors. At the same time, nurturing resilience in pediatric systems might be useful in preventing or reducing children’s internalizing symptoms.

## 1. Introduction

The living conditions of families suddenly and deeply changed during the COVID-19 emergency. Within domestic walls, the educational role of parents has become even more crucial than before. Parents were left alone in taking care of home-schooling their children and also in the management of their children and the home environment, often while engaged in smart-working activities [1]. Houses also became classrooms, workplaces, and play spaces. This situation has significantly increased the risk of experiencing distress and negative emotions in parents and, in turn, the level of parental stress that children are exposed to [2,3]. In a vicious cycle, children absorb their parents’ negativity and stress, inducing behaviors that exacerbate these feelings, thereby endangering the whole family’s wellbeing [4].

The literature related to previous epidemics reported psychological distress in the short term [5,6] and post-traumatic growth and resilience in the long run [7,8,9]. In the same direction, previous studies on the COVID-19 pandemic reported sleep problems and psychological difficulties (e.g., inattention, irritability. clinging, and fears) among children [1,10,11,12,13], and sleep problems, internalizing symptoms, and psychological distress among parents [1,14,15]. Children’s symptomatology in response to prolonged stress results from a combination of protective factors and risk factors of individual/biological and environmental nature. According to the diathesis–stress model, disorders derive from an interaction between a predisposed vulnerability and experienced stress [16,17,18]. The diathesis–stress model (or vulnerability–stress model) tries to explain a disorder or its trajectory as the result of an interaction between predisposed vulnerabilities, diathesis, and stress deriving from life experiences.

In the present study, we aimed to evaluate the role of parental stress and children’s variables (resilience and experience of previous life events) on the development of internalizing and externalizing symptoms in children during the COVID-19 pandemic. It is well known that parental stress has a strong influence on child adjustment [19,20]. Specifically, we operationalized parental stress as reporting low levels of parenting satisfaction and high levels of flooding. Flooding deals with the extent to which a family member’s emotion is perceived as overwhelming and upsetting [21]. It may be experienced by parents when children unexpectedly display negative affect and can induce parents to engage in non-effective parenting, offering the quickest escape from a child’s negative affect but intensifying it in the long run [22,23]. Both parenting satisfaction and flooding are usually related to child adjustment [24,25,26,27]. Significant levels of parental distress might lead to reduced responsiveness, lack of warmth, and a negative view of parenthood, which in turn may lead to an inability to effectively care for children in sensitive ways [25,28,29,30,31].

During the pandemic, parents have been called to face unique psychological difficulties and exceptional levels of stress (e.g., [32]), both of which play a decisive role in shaping parenting practices (e.g., [33]). Consequently, parenting behavior impacts child mental health during stressful life events. There is evidence that parenting behavior is related to a wide spectrum of mental health issues in childhood [34,35,36]. Notably, most of the research outlines that higher mental health difficulties are predicted by negative parenting behaviors, such as hostility and criticism, whereas positive parenting behaviors, like support and warmth, are positively linked with favourable developmental outcomes and negatively linked with child mental health problems (for a review see [37]). The study by Marchetti and colleagues [38] reported that—during the COVID-19 pandemic—child hyperactivity and inattention were negatively influenced by parental verbal hostility. Furthermore, parent hostility and warmth were connected with distinct types of child internalizing and externalizing behaviors [39]. Notwithstanding these premises, little is known regarding whether the mental health of parents and hostile or supportive parenting practices act as contributors to child mental health difficulties from before to during the COVID-19 pandemic.

Beyond parental psychological difficulties and parenting behaviors, a special spotlight should be put on COVID-specific stressors, like isolation, quarantine, and financial difficulties, that families were exposed to, and which might account for both parental and child mental health problems [39,40] along with heightened levels of abusive and neglectful parenting during the pandemic [41,42,43]. Therefore, the present study will consider COVID-19-related stressors and child gender in exploring how parent mental health and parenting behavior could affect children’s internalizing and externalizing symptoms during the pandemic.

Past research consistently found a link between parental stress and children’s/adolescents’ externalizing symptoms [44]. Moreover, as family stress often spills over into parenting behaviors, suboptimal parenting practices have been found to predict children’s externalizing symptoms [45,46]. The role of parental stress in the development of children’s internalizing symptoms is less clear and not always found (e.g., [46]).

There is extensive evidence highlighting the tight relationship between parent and child mental suffering (e.g., [47,48]). Studies highlight that parental anxiety, depression and stress are closely related to both child internalizing and externalizing symptoms (e.g., [47,49]), and this likelihood is meant to increase during stressful periods [32,50,51]. Indeed, during the COVID-19 pandemic, parental mental health problems and stress have been associated with psychosocial concerns in preschool-aged children [52], whereas school-aged children and adolescents were more likely to display internalizing symptoms in the face of depression, anxiety and stress in their parents [39,53]. Thus, further efforts should be spent in researching whether parental psychological disorders and stress during the pandemic might have influenced changes in children’s mental health from before to during the pandemic.

Indeed, understanding whether parental stress during the epidemic period is a specific risk factor for externalizing behaviors or has an impact on internalizing symptoms would be very interesting.

In addition, the potential role played by previous stressful life events in increasing the risk for psychopathology was taken into account. The literature has indeed well-established an association between stressful events and anxiety or depressive symptoms [54,55,56,57,58,59,60,61], as well as a connection between summative indices of stressful life events and angry/acting-out behaviors displayed by children and adolescents [62,63,64,65,66,67]. Interestingly, there is evidence that high levels of parental stress, parental anxious rearing, and dysfunctional parent–child interaction mediate the relationship between stressful life events and the severity of anxiety symptoms in children aged 7–13 years [68]. Moreover, Kim and colleagues [69] reported that exposure to stressful life events fosters the onset of delinquent and aggressive behaviors that, in turn, contribute to reinforcing tough situations and adjustment problems over time.

Finally, we explored the role of resilience abilities as a protective factor against psychopathological symptomatology. Resilience could be defined as a “dynamic process encompassing positive adaptation within the context of significant adversity” [70]. It is an ongoing adaptive process in which protective factors interact with chronic or acute risk factors, bringing positive outcomes [71]. Without a protective factor, higher levels of risk are linked with an increased possibility of a negative outcome. However, when there is a protective factor, it can serve as a mediator of the relationship between the risk and the outcome. Resilient individuals usually display good mental health even if they face serious stress [72]. Resilience changes over time and is shaped by personal strengths and by resources provided in a facilitative environment [73]. Indeed, global resilience can be seen as the result of individual, family related, and community-related resilience factors.

In summary, the purpose of this study was to examine the role of children’s (resilience and previous adverse experiences) and parental (resilience and flooding) factors in predicting children’s psychological symptoms during the COVID-19 pandemic. Building on previous research, which found differences in the association between parental stress and children’s/adolescents’ internalizing vs. externalizing symptoms, we hypothesized a differentiated role of parental stress in those domains. Crucially, we developed our research during two particular phases of the pandemic in Italy: June 2020 (T0), which marked the first return to daily living and working activities after a tightened and prolonged lockdown, and December 2020 (T1), which indicated the second introduction of restriction measures to limit the spread of COVID-19, forcing people to stay at home. A set of questionnaires was completed by parents at two time points. In particular, we collected data on demographic information, adverse childhood experiences and parenting satisfaction at T0; and data on parental flooding, children’s resilience, and children’s psychopathological symptoms at T1.

## 2. Materials and Methods

### 2.1. Participants

A sample of 158 Italian parents (148 mothers and 10 fathers, *M* age = 41 years, *SD* = 5.3 years) was recruited online in the aftermath of the first Italian lockdown period (June 2020) with a snowball sampling. Online recruitment was chosen as it shaped up to be the most suitable way of reaching participants in accordance with the COVID-19 restrictions of the pandemic period. As far as parental occupation is concerned, 41.8% (*n* = 66) of the respondents were full-time employees, 32.9% (*n* = 52) were freelancers, 13.4% (*n* = 21) were part-time employees, 6.9% (*n* = 11) were full-time parents, 4.4% (*n* = 7) were in managerial positions, 0.6% (*n* = 1) were students. As to parental education, 76.2% (*n* = 120) of the participants had a university degree, 20% (*n* = 31) had the equivalent of A-levels, and 3.8% (*n* = 6) had the equivalent of General Certificates of Secondary Education (GCSEs). Concerning geographical distribution, 86% (*n* = 136) of the families were from the North of Italy, 8.9% (*n* = 14) from the Centre, and 5.1% (*n* = 8) from the South. In particular, participants recruited from the Northern Italian regions were distributed as follows: 60.8% (*n* = 96) from Lombardia, 9.5% (*n* = 15) from Piemonte, 7.6% (*n* = 12) from Veneto, 3.2% (*n* = 5) from Emilia Romagna, 2.5% (*n* = 4) from Trentino Alto Adige, and 1.3% (*n* = 2) from Liguria. Participants from the Central Italian regions came from Marche (1.3%, *n* = 2), Toscana (2.5%, *n* = 4) and Umbria (0.6%, *n* = 1), whereas participants recruited from the Southern Italian regions came from Calabria (1.3%, *n* = 2), Campania (1.3%, *n* = 2), Lazio (4.4%, *n* = 7), Puglia (0.6%, *n* = 1), and Sardegna (0.6%, *n* = 1). The remaining subjects did not specify their region of residence (2.5%, *n* = 4). As each parent completed questionnaires for himself/herself as well as for his/her child, we gathered data on 158 children between 5 and 10 years of age (48% boys, mean age = 7.4 years; *SD* = 1.8 years). At the second time point, we collected data from parents of 64 children (45% boys, mean age = 7.58 years; *n* = 1.8 years).

G power analyses show that a sample size of *n* = 77 is enough to obtain a power of 0.80 and a significance level of 0.05 in a stepwise linear regression model with three predictors and with an f2 equal to 0.12.

### 2.2. Procedure

The research project was approved by the Ethics Committee of Sigmund Freud University and was conducted following the ethical standards of the Declaration of Helsinki.

Two time points of assessment were carried out. One parent per family was asked to complete an online survey after being informed about the study, as well as its rationale, scope, methodology and procedures, and having provided their consent to participate (T0). The survey study was advertised via the communication systems of the Sigmund Freud University of Milan as well as social media, and potential respondents were expected to be located in Italy. Inclusion criteria were living in Italy and having a child aged between 5 and 10 years of age.

Six months after the first time-point collection (T0), families were recontacted to complete a new online survey (T1) containing different questionnaires (see below).

### 2.3. Measures

Parents completed the following measures:

Questionnaires at T0:

1. Demographic information. Information on parent and child age and gender, parent education and occupation, and region of residence were gathered.
2. The CYW Adverse Childhood Experiences Questionnaire, CYW ACE-Q [74]—Child version. This clinical screening tool calculates cumulative exposure to Adverse Childhood Experiences (ACEs) in children aged 0–12 years. Parents reported how many experience types applied to their child. It is composed of 17 items: 10 assessing exposure to the original ACEs and 7 to additional early life stressors. Translation into Italian followed published guidelines, including the use of independent back translation. Cronbach’s alpha was 0.69 in the present research, which reflects an acceptable internal consistency.
3. Satisfaction Questionnaire Parenting [75]. The questionnaire investigates parental satisfaction exploring five domains: spouse support, parent–child relationship, parent performance, family discipline and control, and general satisfaction. In the present study, only the subscale “parent–child relationship” was used. It is composed of 10 items with responses on a 4-point Likert scale. Cronbach’s alpha for the parent–child relationship subscale was 0.85 in the present research, indicating a good internal consistency.

Questionnaires at T1:

1. The Child and Youth Resilience Measure—Person Most Knowledgeable version CYRM-PMK [76]. The CYRM-PMK is a parent-report questionnaire measuring (individual, relational, communal, and cultural) resources available to children that may support their resilience. It is composed of 17 items with responses on a 3-point Likert scale. High scores indicate high resilience skills. Cronbach’s alpha was 0.87 in the present research, which corresponds to a good internal consistency.
2. The Strengths and Difficulties Questionnaire (SDQ) [77]. This questionnaire evaluates prosocial behavior and psychological difficulties in children aged 3 to 16 years. It consists of 25 items on a 3-point Likert scale concerning emotional symptoms, conduct problems, hyperactivity–inattention, peer problems, and prosocial behavior. Higher scores on the prosocial behavior subscale reflect “strengths”. Higher scores on the other four subscales reflect “difficulties”, which can be summed to obtain a total difficulties score. Additionally, emotional symptoms and peer problems can be summed to obtain internalizing symptoms score, and conduct problems and hyperactivity–inattention can be summed to obtain externalizing symptoms score. Normative data for the Italian population are available, and the Italian version has good psychometric properties [78]. In the present research, the Cronbach’s alpha for the five subscales ranged between 0.45 and 0.79. Moreover, the overall Cronbach’s alpha was 0.72, which reflects a good internal consistency.
3. The Parental Flooding Scale [26]. This 15-item measure was designed to calculate the degree to which parents perceive their children’s negative affect expressed during parent–child conflicts as impulsive, overwhelming, and confusing. Items are rated from 1 = almost always to 5 = never, with high scores indicating low flooding. Translation into Italian followed published guidelines, including the use of independent back translation. Cronbach’s alpha was 0.96 in the present research, indicating an excellent internal consistency.

### 2.4. Statistical Analysis

Descriptive statistics and correlations among all the study variables were calculated. Then, a stepwise regression analysis to evaluate predictors of the SDQ internalizing and externalizing scores was performed. An alpha level of 0.05 (two-tailed) was adopted as the criterion for statistical significance. Statistical analyses were carried out using SPSS 28.0 [79].

## 3. Results

### 3.1. Descriptive Statistics and Correlations

Externalizing problems showed a high positive correlation not only with internalizing problems but also with low levels of child resilience, satisfaction, and parental flooding (see Table 1). Positive significant correlations were also found among internalizing problems and low levels of child resilience and parental flooding (see Table 1).

### 3.2. Regression Analyses

The results of the regression analysis are shown in Table 2 (coefficients are presented as standardized beta). In the first model, internalizing problems were included as the dependent variable, whereas in the second model, externalizing problems were included as the dependent variable. In both models, the following predictors were included: ACEs, parenting satisfaction, gender and age at T0, and children resilience and parental flooding at T1.

Internalizing problems were predicted only by children’s resilience (step 1).

The regression to predict externalizing problems was performed in three steps: the first predictive variable was Parental Flooding (T1), the second predictive variable was Children resilience (T1), and the third predictive variable was Parental Satisfaction (T0).

## 4. Discussion

The present study aimed to explore which factors explain psychopathological symptoms in children during the COVID-19 pandemic period in Italy.

Overall, our findings revealed that children’s psychopathological symptoms evaluated by parents six months after the first Italian lockdown were significantly predicted by low parental satisfaction self-assessed soon after lockdown and concurrent high flooding and low child resilience. Thus, child resilience seems to be protective against both internalizing and externalizing symptoms, whereas constructs connected with parental stress (both early and concurrent) seem to have an important role in influencing later externalizing symptoms, but not internalizing symptoms.

The first Italian lockdown imposed to contain COVID-19 spread lasted two months and posed unprecedented challenges to family relationships and wellbeing, thereby providing more chances for parent–child conflictual situations due to a prolonged stay at home. Stressful situations increase the likelihood of parents interacting in a less appropriate way with their children, failing in their role of supportive caregivers [1]. Maladaptive parental reactions and ineffective strategies that imply resorting to protective avoidance behaviors of triggering aversive situations (i.e., responding to a screaming child by yelling back or giving in to their demands) might be explained by both individual factors and acquired skills (including resilience) as well as by parent–child exchanges. The present results fit well with recent findings reported by Caputi and colleagues (2021), whereby high levels of flooding were associated with low resilience skills and high novelty-seeking traits in children.

Interestingly, the combined effect of these constructs seems to be observable only on later externalizing symptomatology, as children’s internalizing symptoms were predicted only by low child resilience in the present study. Previous research demonstrated a consistent association between difficult child characteristics and externalizing symptoms [80]. In fact, children with difficult temperaments are often highly irritable and hard to discipline in terms, for example, of limit setting and compliance [81]. Parents of children with a difficult temperament are subjected to higher levels of stress, which, in turn, can induce sub-optimal care. In the same vein, it has been found that both internalizing and externalizing problems contribute to increasing parental stress, with externalizing problems playing a stronger role [82]. Thus, it is plausible that a vicious cycle is triggered that links a specific child’s characteristics and parental stress to externalizing symptoms rather than internalizing symptoms.

Parental stress can be aroused when parents are overwhelmed by parenting demands that they cannot handle [83], and can result in harsher and less consistent parenting behaviors [45]. The pandemic situation has extensively contributed to an increase in such adverse psychological responses. Notably, stressed parents who engage in more negative and coercive parenting behaviors may exacerbate child behavioral symptoms and difficulties in interpersonal family dynamics [84,85,86,87]. However, children with externalizing behaviors may facilitate the onset of more maladaptive coping strategies in parents [88]. Evidence for this vicious cycle can be found in the literature [89]. Several studies reported that parental stress significantly acts as a risk factor for the emergence of children’s externalizing symptoms, specifically hyperactivity/impulsivity, oppositionality, and conduct disorder symptoms [90,91,92,93,94].

In our research, low child resilience shaped up to be one of the most consistent predictors of children’s psychopathological symptoms six months after the first national lockdown. Specifically, low resilience skills predicted high SDQ total difficulties scores. This finding is coherent with the compensatory model of resilience, which is basically rooted in the assumption that being able to adaptively react to adversities promotes good psychological functioning, reducing the impact of stressful life events on daily life [95]. Such a result has important implications for future interventions aimed at reducing the impact on individuals’ wellbeing of big adversities (like a pandemic). Indeed, our findings provide further evidence of the benefits deriving from nurturing lifelong resilience in pediatric systems whenever possible [11,96,97,98].

Unexpectedly, no association emerged between life events and children’s psychopathological difficulties. The reason behind this lack of association could be that our sample was affected by a very low number of life events and was a community sample. The role of stressful life events could emerge in a clinical sample.

## 5. Limitations

For a consistent interpretation of the results, some limitations must be acknowledged. Firstly, notwithstanding the online survey shaped up to be the most suitable tool for reaching the largest sample size in the immediate aftermath of the lockdown period, only 158 parents filled in the questionnaires of the present research project. Moreover, most parents were from Northern Italy and had a high educational level. These sample characteristics limited proper statistical inferences and generalizations of the results to the general population of Italian parents of children aged 5–10 years old. Secondly, the multi-informant approach did not reach an equal proportion of mothers and fathers. Specifically, fathers’ perception of their child’s resilience and emotional/behavioral difficulties was underrepresented here due to the low participation of fathers in the study. The third limitation deals with the absence of direct measures of internalizing symptoms, for which children would have been the best raters. Nonetheless, since our target was children aged 5–10 years, we opted for an online survey to be completed by parents, as younger children would not have been able to complete the questionnaires on their own. Finally, although the present study included two waves of surveys, children’s resilience, psychopathological symptoms, and parental flooding were assessed in the second wave only. Therefore, complete longitudinal analyses could not be performed.

## 6. Conclusions

Notwithstanding these limitations, our research allows us to extend our knowledge regarding factors contributing to the development of internalizing and externalizing symptoms in children during the COVID-19 pandemic. We found that internalizing and externalizing symptoms were predicted by common but also distinct factors. In fact, both internalizing and externalizing symptoms were higher when the child had low resilience skills. Professionals involved in planning future interventions to reduce psychopathological symptoms should be especially aware of this and be sure to include resilience training among featured activities. Moreover, externalizing symptoms were also predicted by high levels of flooding and low levels of parental satisfaction. This suggests focusing on parenting programs to support positive parenting attitudes and behaviors in order to prevent the worsening of children’s externalizing behaviors. The timely delivery of such programs to parents of children with clinically significant levels of externalizing problems is warranted.

Those findings suggest the necessity of planning and defining new psychological programs to support the mental health of children or their parents, especially in the light of the COVID-19 pandemic and the intensification of burdens on families. Fulfilling this urgent need and guaranteeing primary resources for families paves the way for limiting the effects on child mental health. For instance, ensuring support with homeschooling and financial help could contribute to lessening parental stress with inevitable positive reflections on parent–child relationships and on the mental health of both. Additionally, making mental health services more accessible to parents and offering more forms of support will entail reduced pandemic-related stress on parents and their offspring. Parents, for their part, should not be afraid to seek expert help when they sense that their child’s symptoms are becoming unmanageable. If it is impossible to directly access a mental health service, there remains the possibility of requesting consultations and psychological sessions online for parents or for the whole family. Keeping this possibility in mind represents, in many cases, a lifeline and allows parents to feel less alone in dealing with the problem.

Longitudinal research is urgently needed to detect further factors involved in the development of children’s psychopathological symptoms during exceptionally stressful times. Prolonged longitudinal studies with time points spanning several months and years are essential to investigate the long-term effects of COVID-19 and, in particular, pandemic-related mechanisms, like social isolation and parenting behavior, that should be targeted by intervention efforts on child mental health. Moreover, large and representative samples are needed to identify children’s and parents’ risk/protective factors. Wider economic and social inequalities have been intensified by prolonged COVID-19-related restrictions aimed at containing the spread of the virus, resulting in adverse outcomes for the most vulnerable youths. Future studies should be carried out on children with pre-existing risk factors (e.g., learning difficulties, history of mental health problems, lower SES) to better comprehend how to address children with a higher risk of experiencing psychopathological onset.

## Figures and Tables

**Table 1:** Descriptive statistics and correlations among the study variables.

	2	3	4	5	6
1. Life events (0.58 ± 1.24) T0	−0.270 **	0.048	0.025	0.111	0.098
2. Satisfaction (33.23 ± 4.48) T0	-	0.139	0.222	−0.173	−0.332 **
3. Parental flooding (59.63 ± 9.82) T1		-	0.439 **	−0.301 *	−0.584 **
4. Child resilience (30.88 ± 2.55) T1			-	−0.407 **	−0.575 **
5. INT symptoms SDQ (3.77 ± 2.41) T1				-	0.426 **
6. EXT symptoms SDQ (5.53 ± 4.06) T1					-

Note. Significance levels * *p* < 0.05, ** *p* < 0.01, SDQ = Strengths and Difficulties Questionnaire; INT = Internalizing symptoms subscale of SDQ, EXT = Externalizing symptoms subscale of SDQ.

**Table 2:** Linear and hierarchical regression analysis of predictors of internalizing and externalizing symptoms of SDQ.

	Internalizing	Externalizing		
Predictor Variables	Model 1	Model 1	Model 2	Model 3
Parental flooding T1	/	−0.584 ***	−0.410 ***	−0.400 ***
Child resilience T1	−0.407 **		−0.395 ***	−0.356 **
Satisfaction T0	/			−0.197 *
R^2^	0.166	0.341	0.467	0.503
R^2^_adj_	0.152	0.330	0.449	0.479

Note. Significance levels * *p* < 0.05, ** *p* < 0.01, *** *p* < 0.001.

## Data Availability

Due to ethical concerns, supporting data cannot be made openly available.
